# Optical imaging of microfluidic integrated smart hydrogels for research and sensing applications

**DOI:** 10.1016/j.snb.2025.137952

**Published:** 2025-05-22

**Authors:** Saeed Boroomand, Simon Binder, Moritz Leber, Juan Pablo Botero Torres, Jules J. Magda, Florian Solzbacher, Christopher F. Reiche, Lars B. Laurentius

**Affiliations:** a Department of Electrical and Computer Engineering, University of Utah, Salt Lake City, UT 84112, USA; b Department of Biomedical Engineering, University of Utah, Salt Lake City, UT 84112, USA; c Department of Materials Science & Engineering, University of Utah, Salt Lake City, UT 84112, USA; d Department of Chemical Engineering, University of Utah, Salt Lake City, UT 84112, USA; e Blackrock Neurotech Inc., Salt Lake City, UT 84108, USA

**Keywords:** Hydrogels, Microfluidics, Image processing, Glucose sensing, Weka segmentation, Hough transform, Optical sensing platform, Semi-transparent object detection

## Abstract

Smart hydrogels are a versatile class of materials that are attractive for biomedical sensor applications due to their potential biocompatibility and stimuli-responsiveness. However, the integration of these hydrogels into sensors requires development and engineering efforts to optimize the volume phase transition and time response for the respective sensing applications. This work presents an optical evaluation platform for investigating hydrogel swelling properties in a liquid environment with automated flow control. It employs hydrogel features integrated in microfluidic test strips which are easily interchangeable. The evaluation is carried out using an image sensor that records multiple miniaturized hydrogels in parallel. Overall performance is demonstrated by studying the swelling respones of two different hydrogel compositions (based on acrylamide: a polyampholytic glucose-responsive hydrogel as well as a hydrogel optimized for response to ionic strength and temperature) to various external stimuli, which include sodium chloride, temperature, and glucose as well as glucose spiked into serum and blood. The inclusion of relevant body fluids used in diagnostics potentially enables pathways for this sensing platform to be employed at the point-of-care setting.

## 1. Introduction

Hydrogels are formed through chemical and/or physical crosslinking of polymers creating a hydrophilic three-dimensional network. These polymer networks can absorb large amounts of water without dissolving, which is driven by the difference in chemical potential between the inside and outside environments of the hydrogel. This water absorption forms the basis of hydrogels being used as sensing elements. The polymer network can be modified by chemical and biological functional groups creating a specific response to environmental stimuli in the form of a reversible swelling and shrinking of the “smart” hydrogel due to chemical potential differences. Smart hydrogels can respond to environmental variables as well as the environmental concentration of complex biomarkers [1–5]. The reversible volume phase transition (i.e., their water uptake and release depending on the interaction with the stimulus) makes smart hydrogels attractive for sensing applications. Specifically, biomedical sensors can harness the low elastic modulus of hydrogels, comparable to that of soft body tissue [6]. Various methods have been studied in the past to successfully transduce a hydrogel’s swelling state into a measurable signal, including electrochemical [7,8], magnetic [9], acoustic [10,11], mechanical [12], and optical approaches [13–17]. Such hydrogel-based sensors are used for the measurement of blood glucose levels [18], pH value [19], ethanol content [20], ammonia [21], and urea [5,22]. Besides sensor applications, stimulus-responsive hydrogels are also utilized in actuators as they provide a higher energy density compared to piezoelectric actuators, solenoids, and electrostatic actuators [23]. Exemplary hydrogel-based actuator applications include powerless threshold switches [24], microfluidic valves [25], chemo fluidic transistors [26], micro actuator arrays [27], and pumps [28]. Hydrogel-based actuators’ key advantages are their operation in aqueous solutions and the ability to acquire the energy required for the volume-phase transition from their environment. In order to use a hydrogel for a specific application, the stimulus-dependent equilibrium swelling response of the hydrogel must be precisely tailored to the intended use case. The development of hydrogel-based sensors and actuators is often preceded by labor-intensive iterations of hydrogel design consisting of synthesis cycles and (often manual) measurements until the desired stimulus responsiveness is achieved. A common method to determine the swelling response is given by weighing the hydrogel structures with a balance at different swelling states [21] or by optical imaging, often using a microscope camera [29,30]. Weighing requires frequent manual handling steps and can be error-prone due to residual water accumulation on the hydrogel surface. Additionally, it is challenging to perform this technique on miniaturized hydrogel structures or fast hydrogels, as the weighing of small samples entails a high measurement uncertainty and regular withdrawal of the sample from the aqueous measuring solution distorts the swelling time. Optical methods have the advantage that the hydrogel structures can be evaluated without external manipulation between measurements and solution exchanges, enabling the dynamic evaluation of their swelling response. The dimensions of the hydrogels can be extracted with the aid of image processing tools and computer vision (CV) algorithms [30,31]. This method is often limited when the hydrogel image does not generate large contrast compared to the environment. To improve image processing, especially for semitransparent hydrogels, and for medical imaging, usually contrast enhancement agents such as quantum dots, methylene blue, or nano-particles, are added to the hydrogel network that could possibly influence the polymer network and thus affect the hydrogel response [32]. Rückmann et al., developed an optical setup for characterizing the dynamic and static properties of smart hydrogels [30]. In their work, algorithms for the detection of opaque and semi-transparent hydrogel structures using top-view-images were described and the source code was made freely available. While the described experiments were able to successfully demonstrate an evaluation of single-hydrogel-sample’s swelling degrees, the evaluation of semi-transparent structures remained a challenge for the developed algorithm. Additionally, the authors pointed out the importance of improving hydrogel sample placement and adhesion, implementation of an automatic analyte supply, mitigation of optical disturbances due to a meniscus of the liquid in the measurement chamber and establishing a temperature-controlled environment.

Here, an optical evaluation platform based on a microfluidic channel and a computer vision (CV) algorithm is presented. The platform includes automated analyte injection as well as a temperature control setup for the measurement chamber. It is designed to allow the investigation of the volume phase transition for a variety of different smart hydrogels. The hydrogels to be tested are arranged in test-strip like microfluidic channels and are imaged using a commercially available image sensor and a Raspberry Pi. The images of hydrogels are then used to estimate their area over time and therefore evaluate the dynamic swelling properties [33–36]. To enable this estimation a CV algorithm was implemented in ImageJ with the FIJI processing package and Trainable Weka Segmentation. The algorithm consists of three stages: pre-processing, edge detection, and area estimation. Pre-processing involves cropping, grey scale conversion, and contrast enhancement; edge detection utilizes a random forest classifier to segment the hydrogel’s boundaries; and area estimation applies a circle Hough transform to determine the disk radius and estimate its surface area. The simultaneous evaluation of multiple hydrogel features recorded by the image sensor enables a time-efficient measurement procedure of the swelling behavior of smart hydrogels and increases the statistical power of the studies performed with our platform. The platform’s validation study was carried out by using two different types of hydrogels and various sizes to demonstrate its versatility.

## 2. Materials and methods

The hydrogel characterization setup consists of a microfluidic channel with microstructured hydrogels, an automated analyte injection-control system, a camera-based optical read-out, and a CV algorithm that estimates the cross-sectional areas of cylindrical hydrogels (referred to as disks for the remainder of this manuscript) to evaluate their dynamic and steady-state responses. Two different hydrogels were used in this work to show the versatile measuring capability and to demonstrate the cross-hydrogel compatibility of the image evaluation technique. These two hydrogels were: (I) a polyampholytic glucose-responsive hydrogel (GH) as used previously in *in-vivo* experiments for blood glucose measurements [37]; and (II) a dual-responsive hydrogel (DH) with optimized response towards temperature and ionic concentration of sodium chloride salt as used previously in force-compensated hydrogel sensors [38]. All analyte solutions were prepared by either adding glucose or NaCl to the stock solutions. A detailed evaluation and discussion between glucose response of the glucose sensitive hydrogel compared to reference blood glucose measurements in a rat model is presented elsewhere [39]. The detailed preparation process of the two hydrogel formulations can be found in Supplemental Information S2.

### 2.1. Optical evaluation apparatus

**Fig. 1.**
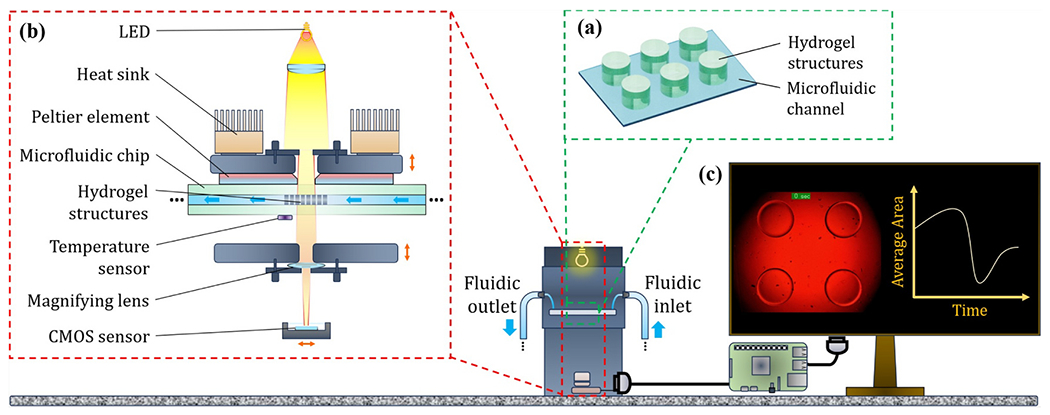
Overview of the optical evaluation apparatus consisting of (a) the microfluidic chip containing the hydrogel structures, (b) the optical image acquisition unit with the optional temperature control setup and (c) the image processing and data visualization unit.

An overview of the optical evaluation platform is shown in Fig. 1. At the core of the design is a microfluidic channel which contains the hydrogel structures to be characterized (see Fig. 1a). The transparent microfluidic channel is fixed by clamps to a disk holder. The channel has fluidic connectors that allow for the connection to a pump system to automatically feed analyte solutions. The optical readout system itself consists of a light source (diameter: 5 mm, operating voltage: 2.1 V, wavelength: 591 nm, Cree Inc., USA), a magnifying lens made of acrylic (diameter: 6 mm, focal length: 9 mm, Edmund Optics Inc., USA), and an image sensor (IMX219, Sony Inc., Japan). The complete arrangement of these components along the optical path is shown in Fig. 1b. The light source is arranged in a transmission topology so that the hydrogel structures on the microfluidic channel are backlit. A light pipe (Dialight Signals and Components, UK) guides the emitted radiation from the LED through to the microfluidic channel. The microfluidic channel holder is designed as a mechanical adjustable stage, which allows the microfluidic channel to be positioned along the optical axis. This enables the adjustment of the focus upon the imaged hydrogel structures. The magnifying lens is supported by a separate adjustable stage so that the position of the lens along the optical path can be modified. This results in tunable magnification of the hydrogel image, which allows us to evaluate hydrogel structures of various diameter (from 2 mm down to 100 μm). Additionally, the position of the image sensor can be adjusted to align its field of view with the hydrogel structures being tested. The channel holder and the optical readout unit are installed in a housing that enables shielding from external light sources. The installation space of this housing without taking the fluidic system into account is 5 cm × 5 cm × 13 cm. To control the temperature of the microfluidic chip, a Peltier element (Laird Thermal Systems Inc., USA) with heat sinks and a fan were clamped to the microfluidic channel holder, as shown in Fig. 1b. Temperature sensors (Pt1000, Innovative Sensor Technology Inc., USA) are attached directly to each microfluidic channel next to the hydrogel structures with heat conductive glue.

The Peltier element, fan, and temperature sensors are operated with a temperature controller (Belektronig GmbH, Germany) enabling the fluid and the hydrogel structures to be maintained at a defined set-point temperature. To enable automated analyte flow, a syringe pump (New Era Pump Systems Inc., USA) is used to draw the fluid from various reservoirs through the microfluidic channel. Electronically controlled pinch valves (Bio-Chem Fluidics Inc., USA) are programmed to block or open fluidic paths enabling to switch between solution reservoirs with different analyte concentrations. The valves are controlled by the Raspberry Pi and switching cycles can be defined via Python. The image acquisition was controlled by the Raspberry Pi as well. However, due to computational constraints of the Raspberry Pi, the CV algorithm was executed on an external PC offline.

### 2.2. Microfluidic channel fabrication

**Fig. 2.**
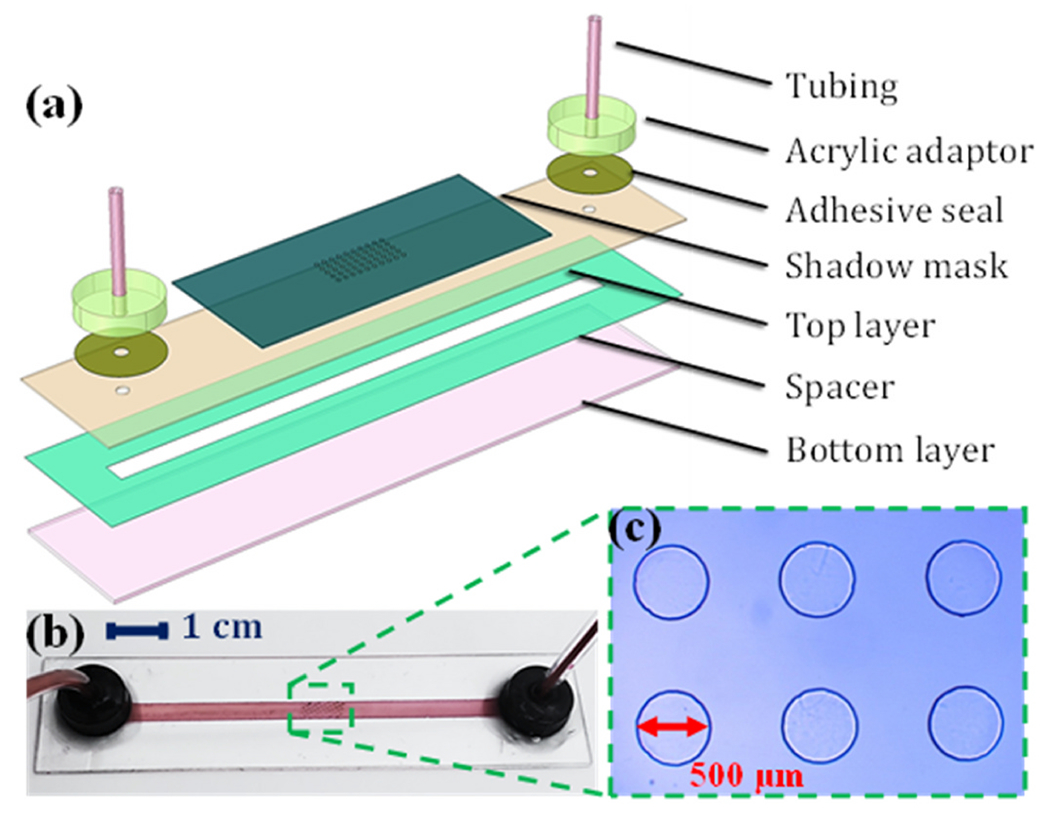
Overview of the microfluidic test channel. (a) Exploded view of the microfluidic channel showing the three layers comprising the test strip and the UV shadow mask, which is used to microstructure a hydrogel within the channel. (b) Image of an actual realization containing circular hydrogel discs in the center. (c) Microscope image of exemplary hydrogel structures in the channel.

The current microfluidic channel design is an updated version of the design used by Leu et al. In this work, the design is optimized for a more reliable long-time operation by improving tube connections and different channel materials, which were selected for improved adhesion properties [8]. The microfluidic channel was fabricated in three layers (see Fig. 2a). The outer dimensions of all layers were 18 mm × 80 mm. The top layer is made of G-UVT (Emco Plastics, thickness: 0.5 mm, Cedar Grove Inc., USA), which was selected for its high UV transmission, laser cutting compatibility, and good hydrogel surface adhesion. Further details on the material selection are discussed in the supplemental section S1. A laser cutter (PLS6.150D, Universal Laser Inc., USA) was used to structure the layers. This layer was fabricated with two cutouts of 0.75 mm radius and 65 mm distance in between as fluidic inlet and outlet. The channel layer was made of a double-sided biocompatible adhesive tape (ARCare^®^ 90445Q, thickness: 0.08 mm, Adhesive Research Inc., USA). It was cut to form a microfluidic channel with dimensions of 3 mm by 68 mm by using a razor blade cutting plotter (Cameo 4, Silhouette America, USA). A polycarbonate layer (thickness: 0.25 mm, McMaster-Carr, USA) served as the bottom floor and was structured with the plotter. Circular tubing adapters were made of acrylic material (thickness: 5 mm) which were laser-cut to have a hole of 3.1 mm for the fitting of the tubing. UV-cured medical-grade epoxy glue (Loctite 3321, Henkel AG, Germany) was used to fix the tubing with the adapters (Tygon PVC Tubing, diameter: 3.2 mm, McMaster-Carr, USA). Fig. 2b shows the realization of the microfluidic channel. The internal dead volume of the test strip fabricated as described is 15.8 μL as derived from the channel dimensions of 3 mm by 65 mm by 0.081 mm. As hydrogels need to be stored hydrated in solution to maintain their functionality, it is recommended to inject fresh solution at least 5 times the volume of chamber, i.e. about 100 μL to fully replace the previous chamber solution content.

### 2.3. Hydrogel patterning

In this work, two different hydrogel types were studied: A glucose-responsive hydrogel (GH), and a dual-responsive hydrogel (DH) with responsivity to salt concentration and temperature as well. The compositions are based in part on previous work and were used with minor modifications. A detailed description for the preparation and composition of the two different pregel solutions is given in the supplemental section S2.

For hydrogel patterning, a UV-mediated hydrogel synthesis was used. The following steps were therefore carried out in a dark room. To create the hydrogel features (see Fig. 2c) inside the microfluidic channel, a pregel solution containing the dissolved educts was injected into the channel via the fluidic inlet with a syringe. A polymer-based photomask (Mylar Mask, DFRRD stack format, Fineline Imaging Inc., USA) with the desired feature pattern was then brought into direct contact with the channel’s UV-transparent top layer and fixed with clamps (see Fig. 2a). In this work, shadow masks with circular openings of 150 μm, 300 μm and 500 μm in diameter were used, which resulted in circular hydrogel disk of 81 μm height. The spacing between the disks was 150 μm, 300 μm and 500 μm, respectively. The pregel solution was UV-exposed through the photomask using a collimated UV light source (Omnicure S1000, 365 nm center, 25 ± 2 mW/cm^2^, Waltham Inc., USA) to selectively initiate a free radical polymerization resulting in the formation of the hydrogel features. Exemplary hydrogel features created this way are shown in Fig. 2c. The exposure time varied with the type of hydrogel. After the exposure step, the channels were flushed to remove the uncured pregel solution. Depending on the hydrogel type, either phosphate-buffered saline solution (1XPBS) or deionized (DI) water was used for this step. All the hydrogel types were conditioned prior to use by at least three cycles (24 h), going from 1XPBS to ¼XPBS and back to 1XPBS. This process is required to obtain a reproducible swelling behavior since, for example, shorter polymer network segments must be broken up first, and other polymer chains have to align themselves [23]. To assess the uncertainty in the hydrogel size distribution of this fabrication process, a microfluidic channel was fabricated with hydrogels of a target diameter of 300 μm. The diameter of 15 randomly selected hydrogel structures out of 100 total structures was measured under the microscope. The average hydrogel disk size was found to be (304 ± 4) μm. It was observed that hydrogel structures partially at the mask edge polymerized incompletely. Therefore, hydrogel structures located in the center of exposure were used for the studies performed in this work.

### 2.4. CV algorithm

**Fig. 3.**
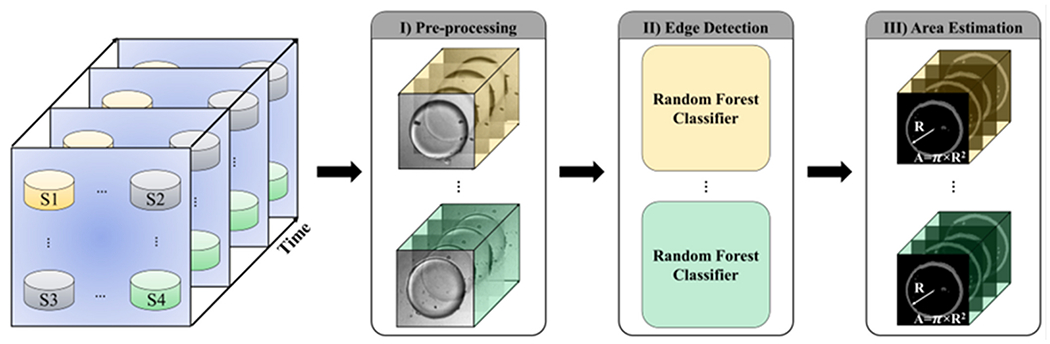
Computer vision processing pipeline consisting of three stages: I) pre-processing where the image is cropped, converted to greyscale and contrast enhanced; II) edge detection in which a custom random forest classifier is applied to each hydrogel disk; and III) area estimation where a circle Hough transform is used to estimate the radius and location of the hydrogel disk and its surface area is calculated.

To evaluate the volume phase change of the hydrogel disks within an experiment, a series of images were acquired using a Raspberry Pi and a complementary metal-oxide semiconductor (CMOS) camera sensor. The image acquisition frequency was determined by the response dynamics of the hydrogel type studied. A CV algorithm was devised to process the resulting image series and estimate the surface area of the hydrogel disks. The semi-automated algorithm was implemented through ImageJ, an open-source image processing software, and utilized complementary plugins including the FIJI processing package [40], UBC Vision Sciences, and Trainable Weka Segmentation. The CV algorithm is composed of three main processing stages: pre-processing, edge detection, and area estimation (as shown in Fig. 3).

#### 2.4.1. Pre-processing

The optical approach allows us to simultaneously record the phase volume transition of several hydrogel disks. In this study, the CV algorithm considered single hydrogel disks individually. Thus, in the pre-processing stage the images were first cropped to include a single hydrogel disk. The cropped images were then converted to greyscale format, as the color information is irrelevant for determining the swelling state of the hydrogel disks. To reduce the computational burden of the CV algorithm the greyscale images were downscaled to a quarter (approximately 500 ×500 pixels, variations in size are due to the manual cropping procedure). As the final step on the preprocessing stage the contrast on the images is enhanced using contrast limited adaptive histogram equalization (CLAHE), which enhances the contrast of the images locally to account for uneven lighting conditions.

#### 2.4.2. Hydrogel edge detection

To detect the edge of the hydrogel disks a fast random forest (RF) was used, which is a multi-thread version of RF and a pixel-based binary classifier. This algorithm applies a series of filters and transformations to represent each pixel as feature vector, then the RF classifies the features either as background or edge using a pre-defined number of decision tree predictors. The classifier was implemented using the Trainable Weka Segmentation plugin, and the features selected were: “Gaussian blur”, “median”, “variance”, “mean”, “entropy”, and “neighbors”. As RF classifiers are a supervised learning technique, a custom model was trained for each hydrogel disk evaluated. The training was performed using manually annotated images. The annotation process was facilitated through the graphical interface of ImageJ, that allowed to outline the edge of the hydrogel and select exemplary background regions. The background regions selected for each training image were carefully chosen to include possible artifacts such as bubbles and debris in the optical path. This selection was made to reduce the probability of misclassifying such artifacts as part of the hydrogel’s edge. A detailed description of Weka’s RF classifier, its training procedure, and parameters, can be found elsewhere [34]. It is important to note that even though a custom model was trained for each hydrogel disk, the training parameters and the model’s topology remained constant (see Table S-3). Due to the labor-intensive nature of the training process, the number of training images needed to achieve robust performance of the CV algorithm was analyzed. Details of the analysis are presented in the supplemental section S3. The custom models for each hydrogel disk were trained with three calibration images (with low, mid and high hydrogel swelling degree) and were subsequently used for inference of all the pre-processed images of that particular disk, obtaining images containing the segmented hydrogel disk edge.

#### 2.4.3. Area estimation

In the final processing stage, the area is estimated using a circle Hough transform (UBC Vision Sciences) which allows to extract the radius and center coordinates of a binary image. To implement this transform, the image was first upscaled to its original size (approximately 1000 × 1000 pixels). This is done to reduce the error associated with the radius estimation. Then, a median filter is applied, and the image is binarized, to remove high frequency noises. All binarized images, containing the edge of the hydrogel disk, were then processed with the circle Hough transform. This transform uses previous knowledge like the expected number of circles in the image and a range of expected radius, to reduce the search space. Finally, the area is calculated using the estimated radius. Table S-3 contains the list of parameters used for the transform.

### 2.5. Measurement procedure

Measurements were performed for each of the two types of hydrogels. Depending on the hydrogel to be characterized, specific measurement solutions were used. Glucose responsive hydrogels are of much interest due to their improved stability and reliability compared enzyme-based sensors and the potential biocompatibility that hydrogels have. We used a known glucose-sensitive hydrogel formulation, which works based on reversible binding of phenyl boronic acid (PBA) functions with a diol-containing moiety such as glucose in two reactions [41]. Glucose binds with two hydroxyl groups to a charged boron formation. In a second step, glucose binds to an amine group provided by N-[3-(N,N-Dimethylamino) propyl]acrylamide. Therefore, the binding of glucose not only increases the crosslinking in the hydrogel but also results in no additional charges. These two effects result in hydrogel shrinking. Glucose is unique in that it contains two planar diols, which can form the reversible crosslinks and thus making this class of glucose sensitive hydrogels more specific [42,50]. For the characterization of the glucose-responsive hydrogel (GH), solutions with glucose concentration in the range of 125 μM to 30 mM were prepared, both in 1XPBS solution as well as in 1:10 diluted serum solution with 1XPBS (Pooled human AB serum plasma, ISERAB 100 mL, Innovative Research, USA) and in 1:10 diluted human blood with 1XPBS (Age: 50, Gender: M, Innovative Research, USA). Testing the glucose response in a biological medium is intended to demonstrate the versatility of the presented methods and evaluation algorithm for the characterization of hydrogels in physiologically relevant fluids. Glucose in powder form was added to the biological sample solutions. The blood stock solutions received from the supplier were devoid of glucose due to glycolysis. The pooled serum solution, however, contained a known amount of glucose as measured and documented by the Innovative Research. For the dual-sensitive hydrogel (DH), sodium chloride solutions were diluted ranging from 1 mM to 1000 mM. The temperature stimulus for the DH hydrogel was adjusted in the range between 15 °C and 40 °C by controlling the set-point temperature with the temperature controller. Solutions were filtered before injection (UNIFLO 25 syringe filters, Whatman, USA) and were additionally processed by argon purge and vacuum degassing for 5 min each to avoid bubble formation. The flow rate for all experiments was kept at 1.2 mL/h for the microfluidic channels (GH and DH hydrogels). A flow rate 10–100 times higher was used for flushing and cleaning of the channels, which is done before the beginning of each study. For dynamic swelling evaluations, images were taken with a rate of 2 images/min for DH and 150 μm in diameter GH disks, and 0.5 images/min for others. The presented swelling data was normalized to the surface area of the highest swelling degree. Data points in the diagrams represent the average normalized disk area in percent from a number *n* of hydrogel disks. The error bars represent the accompanied standard error of the average normalized disk area.

## 1. Results & discussion

### 3.1. Verification of CV algorithm

To assess how reliably the CV algorithm estimates the cross-sectional areas of a hydrogel disk, a selected image data set (*n* = 15) of a single hydrogel disk (300 μm diameter mask) was evaluated manually for comparison. This was done by manually fitting a circle segment to the hydrogel outlines in each image using ImageJ’s “free selection”, “wand”, and “ROI manager” tools. The same set of images used for manual annotation was evaluated by the CV algorithm.

Table 1 shows the diameters of the hydrogel top surface for three different swelling states based on varying glucose concentrations in 1XPBS solution. The CV estimation is in good agreement with the manual evaluation. There is only a slight overestimation of less than 1 % when using automated evaluation. A paired two-tailed T-test was conducted on the fairly normally distributed dataset and it was found that there is no significant difference between the disk area evaluated by any of the methods. Thus, it can be concluded that the classification model derived through training is sufficient for the automatic segmentation of the images.

Additionally, to further evaluate the performance of the CV algorithm across different hydrogel disks, the Jaccard similarity coefficient was calculated. This performance metric evaluates the overlap between the predicted area and the manually annotated images. The evaluation was performed using the GH swelling study. 30 random images were drawn from this data set for each of four studied hydrogel disks for the experiment. The Jaccard coefficients were calculated at 98.0 ± 0.8 %, 99.0 ± 0.9 %, 98.0 ± 0.6 %, 97.0 ± 0.6 %, indicating a good match between manual and automated analysis. A more detailed description of the performance analysis for the CV algorithm is presented in the Supplemental Information section S3.

### 3.2. Glucose-responsive hydrogel measurements

**Fig. 4.**
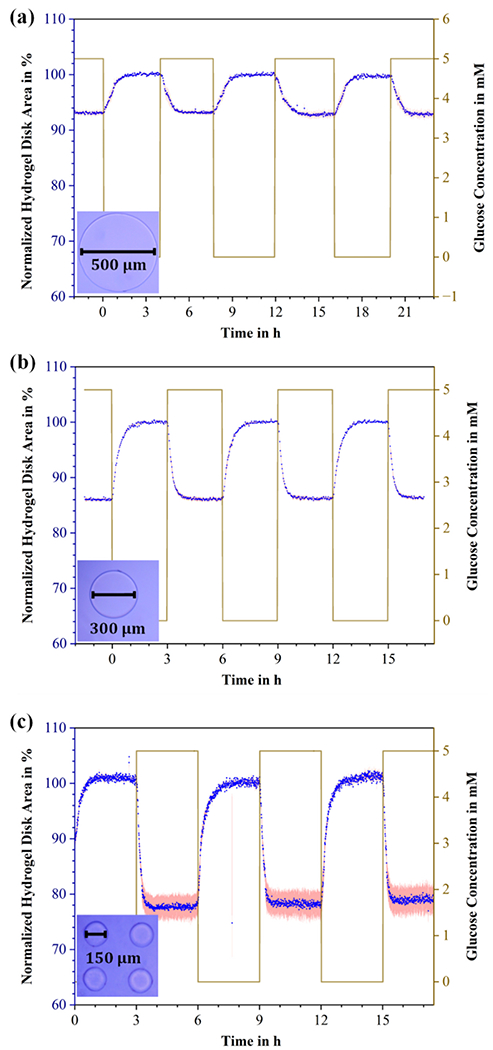
Time-dependent swelling and shrinking of the cross-sectional area of hydrogel disks with changing glucose concentrations for hydrogel disks with (a) 500 μm diameter (*n* = 4), (b) 300 μm diameter (*n* = 3), and (c) 150 μm diameter (*n* = 4). The red shaded area represents the associated standard error.

In the first experiment, the influence of different hydrogel diameters on the measurement procedure and the hydrogel swelling dynamic behavior was investigated. For this experiment, microfluidic channels with GH hydrogel disks of 500 μm, 300 μm, and 150 μm diameters were fabricated. Fig. 4 shows the swelling response over time for each of the three hydrogel dimensions in response to cyclic changes between 1XPBS and 5 mM glucose dissolved in 1XPBS. The experiments were carried out in three cycles to demonstrate their repeatability. Please note that the swelling data shown in Fig. 4a originates from two separately fabricated microfluidic channels. The average change and standard errors were derived by combining the data from hydrogel disks of the first channel (*n* = 3) and second channel (*n* = 4). The swelling data from Fig. 4b and c originate from multiple disks inside a single channel for each experiment. From the time-resolved measurements it can already be seen, that both the overall top surface area change as well as the response time are related to the fabrication diameter. Table 2 shows the average swelling and shrinking response times **τ_90_**, which corresponds to the characteristic time until 90 % of the steady-state value is reached. From Table 2, it can be seen that the response times decrease with the diameter of the hydrogel disks. Decreasing response time with a shorter critical dimension of a hydrogel, in this case the diameter of the hydrogel disks, is commonly known and, thus, observable with the presented optical evaluation platform here as well [8,11]. Please note that the response time for swelling can be considered as the recovery time for the hydrogel if more than one sample is supposed to be measured.

While enhanced response dynamics with decreasing hydrogel dimensions is an advantage for optical evaluation methods, it could be a limiting factor for other characterization and sensing methods. For instance, where the hydrogel has to perform mechanical bending work to generate bending strain [43,44]. In such case, the bending radius of cantilever-based mechanical hydrogel sensor coated with a thin hydrogel layer would decrease and, thus, reduce the sensor’s sensitivity. The results in this work show the feasibility of the evaluation of hydrogel dimensions as small as 150 μm in diameter. The compatibility and training-based evaluation with disks of such small dimensions is advantageous, as this keeps the characterization times of hydrogels to about 15–30 min, making it possibly suitable for a clinical point-of-care setting. Considering the turnaround times and small dead volume of the test cartridges (<20 μL), we believe it is possible to introduce body fluids to the sensor via injection or modified capillary action methods and potentially have a short-lag glucose sensor which can function within the range of diagnostics requirements [45].

From Fig. 4, it can also be seen that the overall hydrogel swelling response changes with the fabrication diameter, i.e., from approx. 7 % area change (for 500 μm diameter disks) to about 14 % area change (300 μm diameter disks) to about 25 % area change (150 μm diameter disks). Given that all hydrogel disks had the same thickness, the aspect ratio of hydrogels varies with the diameter. It is expected that a disk’s top surface is less constrained in swelling by the adhered bottom face if the disk has a higher aspect ratio. This might lead to improved swelling and shrinking responses. The smallest hydrogel disk diameter considered in this work of 150 μm results in the strongest swelling response and shortest response time. However, there is more variation in the swelling response between individual hydrogel disks of this size, which can be seen from the larger error bars for disks with 150 μm diameter. Disks with diameters of 300 and 500 μm, on the other hand, did not deviate as much from each other in their swelling behavior. Therefore, hydrogel disks with a diameter of 300 μm were primarily used for the investigations performed in this work, as they exhibit a good trade-off in terms of response time, swelling response, and disk variability. Hydrogel disks with dimensions below 100 μm were not achievable in this work due to the limitations of the non-ideal properties of the collimated UV light source and the limited resolution the printed photomasks. However, using higher grade microfabricated chromium masks and more complex collimated light sources such as used for typical microfabrication methods can improve the outcome further.

**Fig. 5.**
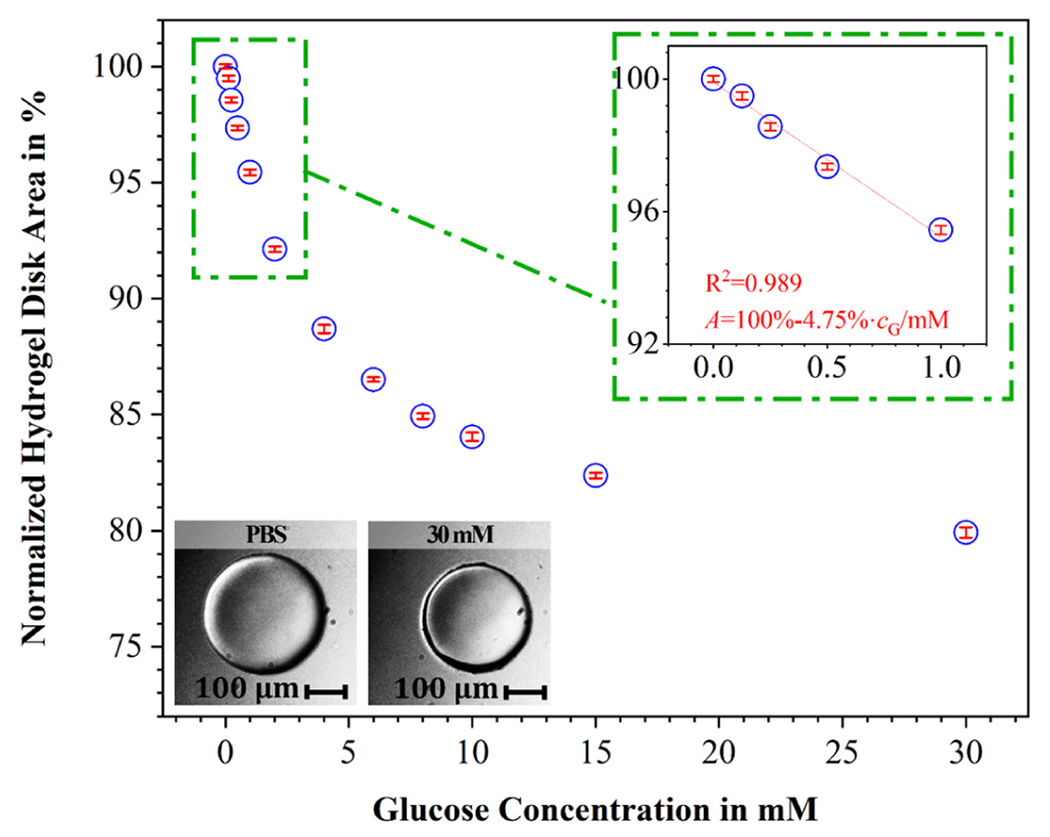
Steady-state values of 300 μm hydrogels’ cross-sectional disk area normalized to the disk area in 1XPBS. Swelling data from the images of each step’s last 10 min were averaged. Error bars represent the standard deviation when considering the swelling response of all three evaluated hydrogel disks in the field of view of the camera sensors.

In an additional experiment, the response to a variety of glucose concentrations in 1XPBS was studied. The glucose concentration increments were chosen to be in the physiologically relevant range. Fig. 5 shows the swelling response for three half cycles of glucose variation and three hydrogel disks (300 μm in diameter) obtained with the optical evaluation setup. Data points in the figure show the corresponding steady-state values normalized to the disk area in 1XPBS. The measurements show a significant shrinking of the hydrogel structures with increasing glucose concentration, which is consistent with the results of previous work on hydrogel-based glucose sensors [11]. No significant hysteresis during swelling and shrinking cycles was observed. The glucose-sensitive hydrogels responded to glucose concentrations as low as 125 μM and up to 30 mM. The limit of detection (LOD) can be given as 88 μM. It was calculated as the intersection point on the axis of the calibration curve where three times the standard error of blank was subtracted from the blank value on the linear local fitting curve. The relationship between the hydrogels’ top surface size and the glucose concentration appears to be non-linear for glucose concentrations above 1 mM. The highest sensitivity is to be found between 0.5 mM and 4 mM of glucose concentration, which corresponds to the typical glucose levels present in the condition of hypoglycemia [46]. The time-resolved data for this experiment is given in the Supplemental Information section S4. Images for one hydrogel disk sample at the end of each test step are presented in the Supplemental Information section S7.

### 3.3. Measurements in physiologically relevant fluids

**Fig. 6.**
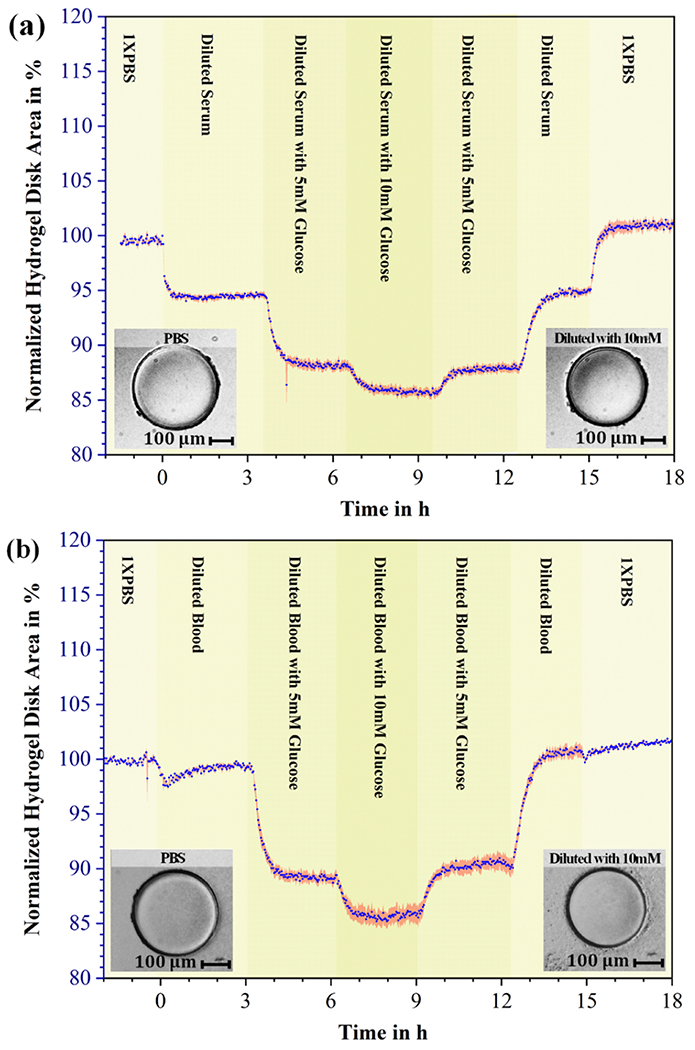
Glucose response of GH in human serum and blood. Time response of glucose-responsive hydrogel disks (diameter: 300 μm) towards different concentrations of glucose in (a) diluted serum (n = 4) and (b) diluted blood (n = 3) as well as the baseline area in 1XPBS. The red shaded area represents the associated standard error at each time point.

In this experiment, a microfluidic channel containing glucose-responsive hydrogels with a diameter of 300 μm was exposed to diluted serum as well as diluted blood with distinct levels of glucose. The swelling responses of four (serum) and three (diluted blood) hydrogel disks within the microfluidic channel are shown in Fig. 6. To compensate for the fact that a small number of proteins and cells might accumulate at the hydrogels’ edges, serum and blood experiments were analyzed by processing the area of the internal edges of the hydrogel disks. In both solutions, the hydrogel disks shrink with increasing glucose concentration, thus showing pronounced responsiveness towards glucose within serum and blood.

From Fig. 6 the hydrogels shrunk in response to the diluted serum solution due to the residual amount of glucose being present (5.4 mM diluted by 1/10, resulting in 0.54 mM). However, this was not observed with diluted blood as the glucose is consumed by the red blood cells during disk storage. The measurements shown demonstrate that the optical platform and evaluation algorithm can also be operated with physiologically relevant fluids as complex as blood, possibly opening its use as a measurement instrument in clinical point-of-care settings as well. Images for one hydrogel disk sample at the end of each test step are presented in the Supplemental Information section S8 and S9.

### 3.4. Dual-responsive hydrogel (DH)

**Fig. 7.**
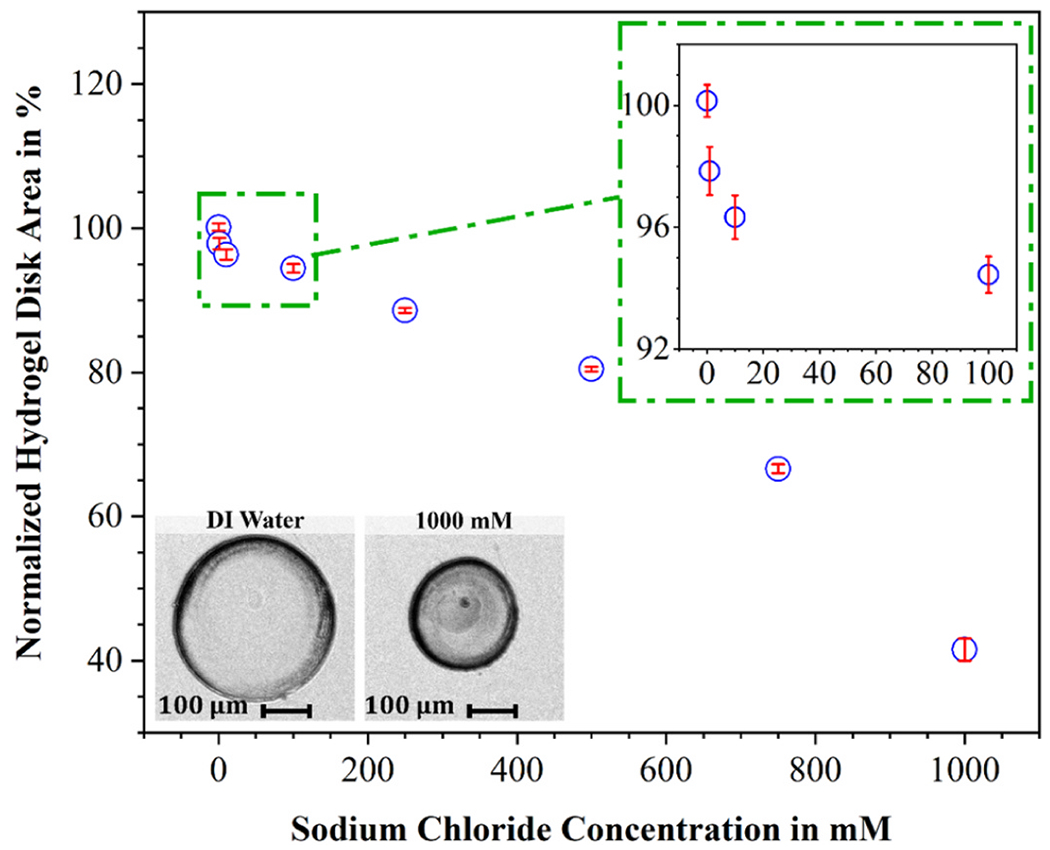
Steady-state swelling data for three hydrogel disks (diameter: 300 μm) as a function of sodium chloride concentration. The swelling data and the standard error at each step were averaged from the images of three disks during the last 10 min.

**Fig. 8.**
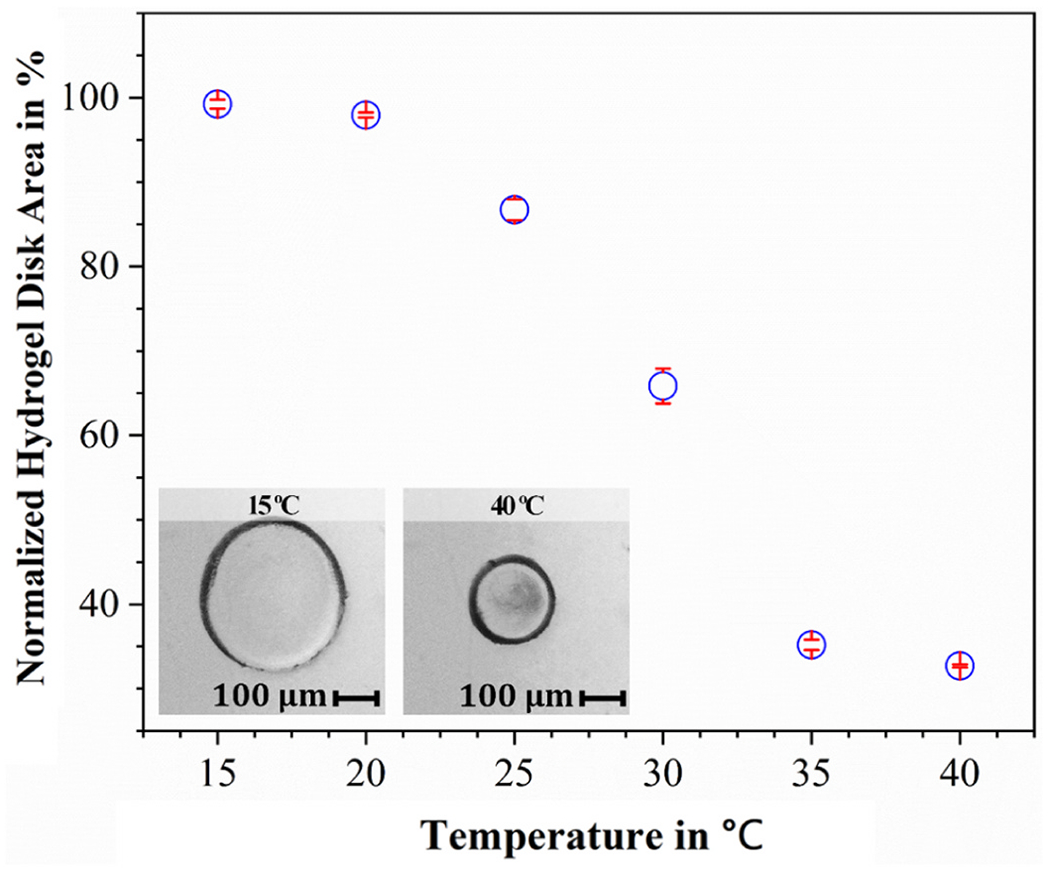
Steady-state swelling data of hydrogel disks (diameter: 300 μm) inside a microfluidic channel as a function of temperature. DI water was passed through the channel while adjusting the temperature (uncertainty: ±0.1 K). The change between temperatures takes place in less than 15 s, due to the active heating and cooling by means of the Peltier element. Error bars represent the standard error for three hydrogel disk measurements during the last 10 min of each analyte solution cycle.

The optical evaluation is intended to be highly versatile regarding various stimuli and to serve the characterization of many different hydrogels for a wide variety of applications. To underline this, a multistimuli responsive hydrogel was investigated, which is expected to show pronounced responsiveness to temperature as well as salt concentrations [49]. Such a hydrogel was successfully created in the microfluidic channel and characterized in the optical measurement setup. Its thermo-responsiveness is based on the presence of NiPAAm side groups in the polymer network. Linear PNiPAAm polymer strands are known to exhibit a lower critical solution temperature (LCST) behavior, i.e., the solubility rapidly changes at the LCST of 32 °C. When PNiPAAm is crosslinked to form a hydrogel, the LCST behavior causes a temperature-dependent uptake of water into the polymer network instead. The additional responsivity towards ions in a surrounding solution is caused by acidic side groups incorporated into the hydrogel’s polymer backbone. The dissociated acid groups carry negative charges, which drive the polymer network apart by means of electrostatic interactions. The concentration of counterions in the surrounding solution influences the dissociation and thus the degree of swelling of the hydrogel. Fig. 7 shows the swelling behavior of the hydrogel disks as a function of sodium chloride solutions, while Fig. 8 shows the hydrogel’s temperature response. Corresponding images for one hydrogel disk sample at the end of each test step are presented at the Supplemental Information section S10 and S11.

From the diagrams, it can be seen that the hydrogel exhibits both the desired salt responsivity and temperature responsivity. The salt responsivity is well-pronounced over a range from 0.1 M to 1 M. This somewhat differs from previous work with this hydrogel type, where salt responsivity was to be found over a much broader concentration range down to 0.001 M [47,49]. The limit of detection in this work is 10 mM and was calculated as described above. The temperature-induced swelling curve reflects the typical swelling behavior of a NiPAAm-based hydrogel with an inflection point close to the LCST (see Fig. 8). If acid groups are additionally present in the hydrogel, however, one would expect the swelling response to be pronounced over a broader temperature range up to approx. 50 °C, in line with previous work. In the measurement shown in Fig. 6, though, the responsivity is only pronounced up to approx. 35 °C. Both the slightly different swelling behavior towards salt concentrations and temperature compared to previous work is possibly due to the fact that the synthesis parameters were slightly changed. For instance, instead of a thermal initiation of polymerization, a UV initiator was used in this work, and the polymerization of the hydrogel was carried out under UV exposure. This may affect the cross-linking density and, thus, alter the swelling behavior. What should be emphasized though with this type of hydrogel is the in general much more pronounced change in swelling degree compared to the glucose-sensitive hydrogels. With dual-responsive hydrogel, a shrinking down to 30 % of the original dimensions is achieved. While no detachment of the hydrogel disks from the bottom layer of the channel occurred during the characterization with salt concentrations, this was observed for some hydrogel disks in the high-temperature range above 40 °C. It appears that not only the amount of shrinkage is crucial for adhesion to the microfluidic chip’s bottom layer but also the type of stimulus. It can be concluded that hydrogel disks can be safely operated down to 40 % of their original top surface diameter when applying salt concentrations and up to temperatures of 40 °C for the temperature stimulus. It should also be noted that the temperature response of the hydrogel occurred more quickly than the salt response, which is consistent with previous work on such dual-responsive hydrogels [47]. Time-resolved measurements for this hydrogel are shown in the supplemental section S5. These experiments demonstrate that the presented platform can as well serve as a versatile tool for the investigation of more complex swelling behaviors, as it occurs in multi-responsive hydrogels. In particular dual-responsive hydrogels, such as the presented one, are utilized in advanced sensing techniques, such as the compensation measurement method for faster sensor responses [38,48,49]. The platform can thus be helpful for rapid prototyping of such hydrogel formulations. If the platform directly were to be used to measure salt concentrations, the temperature of the hydrogel would be fixed using the platform’s internal temperature control unit to suppress the temperature cross-sensitivity. As an alternative, other hydrogels could be synthesized in the channel that are either salt-sensitive (i.e. omitting the NiPAAm monomers during synthesis) or temperature-responsive (i.e. omitting the acid monomers during synthesis).

## 4. Conclusions

In this work, new methods and tools for the optical characterization of hydrogels in a microfluidic channel were presented. The developed optical evaluation platform adds to previous setups by its small footprint, easily interchangeable hydrogel test strips, automation of fluid injection, batch image processing and the parallel analysis of multiple hydrogel features within a single test strip. The training to obtain a custom model for a new hydrogel type inside a microfluidic channel required three calibration images at different swelling states, allowing the model to be derived in less than an hour on a personal laptop. Once the model has been derived, evaluation of the hydrogel swelling state from a single time step requires a few seconds only. As this uses only existing ImageJ plug ins, no programming skills are required. Thus, this makes the presented method easy to implement and use. Due to the individual calibration training for each hydrogel sample structure, this evaluation method is expected to be more robust against changing light conditions or other external influences like bubbles and contaminations than previous optical evaluation methods. Furthermore, the optical evaluation platform consists of low-cost components, which should allow other research groups that frequently characterize hydrogels to easily replicate and adapt it to their own purposes. The capability to characterize different types of hydrogels in different application scenarios was demonstrated by measurements with two different hydrogels. It can be concluded that the presented evaluation platform, thus, can be helpful in the hydrogel design phase when novel hydrogels with a specific swelling behavior or degradation rate are to be developed. The versatility of the described approach is further underlined by the fact that temperature control of the hydrogel disks and compatibility with serum and blood as analyte fluids were demonstrated. This could not only allow hydrogel characterization, but also sensing of various physiologically relevant stimuli, thus making this evaluation platform possibly attractive for point-of-care applications, either in laboratories or clinical settings. Instead of large test containers the volume of the measurement chamber is 20 μL allowing fast tempering of the disks and reduced analyte consumption for expensive solutions. The microfluidic hydrogel test strips are easily interchangeable. It is envisaged that hydrogels responsive to different stimuli can be prepared in a single channel, e.g., by sequential UV crosslinking of different hydrogels or that several channels, each with different hydrogels, are all evaluated at the same time within one field of view of the image sensor. This would make it possible to detect a wide variety of components, e.g., in blood samples, with one single measurement setup and measurement run. Having additional hydrogels with other responsiveness within the field of view might also be attractive to increase selectivity of the measurement principle, since hydrogels are known for their pronounced multistimuli responsivity. The design of such microfluidic test strips and their characterization for point-of-care applications in hospitals and medical practices is the subject of future research.

## Supplementary Material

1

## Figures and Tables

**Table 1:** Comparison between manual and automated evaluation,

Glucose	Manual	CV Estimation
0 mM*	(314.0±0.8) μm	(313.0±0.4) μm
5 mM*	(294.5±0.9) μm	(295.0±1.0) μm
30 mM*	(281.3±0.8) μm	(281.3±0.5) μm

* denotes a P < 0.05.

**Table 2:** Response time (τ_90_) of hydrogel disks with different fabrication diameters.

Diameter	500 μm (n = 7)	300 μm (n = 3)	150 μm (n = 4)
Swelling τ_90_ in min	(70±*3*)	(52±2)	(33±1)
Deswelling τ_90_ in min	(50±*3*)	(28±2)	(14±1)

## Data Availability

Data will be made available on request.

## Appendix A. Supporting information

Supplementary data associated with this article can be found in the online version at doi:10.1016/j.snb.2025.137952.
