# Supplementary material for: Optical imaging of microfluidic integrated smart hydrogels for research and sensing applications
Source: Sens Actuators B Chem. Author manuscript; Available in PMC 2026 Jul 2. (PMC13322282; doi:10.1016/j.snb.2025.137952)
Supplement: 1 [file NIHMS2175145-supplement-1.docx]

**Supplementary Information**

Optical imaging of microfluidic integrated smart hydrogels for research and sensing applications

Saeed Boroomand^1^, Simon Binder^1^, Moritz Leber^5^, Juan Pablo Botero Torres^2^­, Jules J. Magda^4^, Florian Solzbacher^1,2,3^, Christopher F. Reiche^1^, and Lars B. Laurentius^1*^

^1^Department of Electrical and Computer Engineering, University of Utah, Salt Lake City, UT, 84112, USA.

^2^Department of Biomedical Engineering, University of Utah, Salt Lake City, UT, 84112, USA.

^3^Department of Materials Science & Engineering, University of Utah, Salt Lake City, UT, 84112, USA.

^4^Department of Chemical Engineering, University of Utah, Salt Lake City, UT, 84112, USA.

^5^Blackrock Neurotech Inc., Salt Lake City, UT, 84108, USA.

*Corresponding author e-mail: Lars.Laurentius@utah.edu

**S1. Material Selection for Microfluidic Channel Design**

The microfluidic channel design involved the selection of UV transparent materials. Different materials were tested both as the top and as the bottom layer of the channel. Tested material stacks are listed in Table S1. Initially, polycarbonate sheets were used as both the top and the bottom layer material which resulted in mechanically flexible channels. The high degree of flexibility made sealing the inlet and outlet ports challenging. Thus, as a second approach an acrylic sheet was used as one of the two layers (Stack #1). The acrylic sheet was easy to laser cut and the resulting channel was not bending during handling. However, with flow rates higher than 1.2 mL/h hydrogel disks partly sheared off from the acrylic surface. Moreover, a glass slide as a bottom layer was found to be unsuitable for long studies, as the channels were prone to leakage due to weak sealing between adhesive tapes and glass in PBS solutions (Stack #2). PTFE was used in one variation to have a low friction material as the bottom layer to avoid adhesion of hydrogel disks carried by the top layer (Stack #3). While PTFE tapes showed much reduced adhesion towards hydrogels the glass substrate still caused leakage. Finally, an approach using a UV transparent, laser cutting compatible and mechanically strong layer of G-UVT material resulted in a microfluidic channel with satisfying results regarding handling, hydrogel adhesion and sealing (Stack #4). Details about the UV transparency as well as an adhesion study for the different stacks are given in Table S1. For the adhesion study, channels were made and peeled off manually at a shallow angle, slowly to observe hydrogel disk adhesion preference to different surfaces. GH hydrogels were used in this study.

**Table S1. UV transparency and hydrogel disk adhesion results for different materials**

| Microchannel Stack Structure | Top Layer  (UV Transmission) | Bottom Layer  (UV Transmission) | % of Disks Adhering to Top Layer After Peel-off | % of Disks Adhering to Bottom Layer After Peel-off |
| --- | --- | --- | --- | --- |
| Stack #1 | 0.25 mm-thick polycarbonate (90%) | 0.5 mm-thick acrylic (0.5%) | 97% | 3% |
| Stack #2 | 0.5 mm-thick G-UVT (94%) | 1.0 mm-thick glass slide (91%) | 0% | 100% |
| Stack #3 | 0.25 mm-thick polycarbonate (90%) | 0.1 mm-thick 3M^TM^ 5490 PFTE tape on glass (58%) | 100% | 0% |
| Stack #4 | 0.5 mm-thick G-UVT (94%) | 0.25 mm-thick polycarbonate (90%) | 87% | 13% |

**S2. Hydrogel preparation**

*Glucose-responsive hydrogel (GH):* The glucose-responsive hydrogel is based on immobilized phenylboronic acid (PBA) in the polymer matrix as presented in previous work [8], [50]. The GH pregel solution was prepared as follows. A 1 mM buffer solution of 4-(2-hydroxyethyl) piperazine-1-ethane sulfonic acid (HEPES, Sigma-Aldrich, USA) was prepared with DI water, and its pH value was adjusted to be 8. Then, 57.3 mg of 3- acrylamidophenylboronic acid (3-APB, Achemo, China) was dissolved in 261 µL dimethyl sulfoxide (DMSO, Fisher Scientific, USA) followed by vrtexing for 20 s. Subsequently, 711 µL of a 30 wt% solution of acrylamide (Aam, Poly- sciences Inc., USA) in HEPES buffer was added to the solution vial and vortexed for 10 s. Then, 579 µL of a 2 wt% N, N’- methylene bisacrylamide solution (BIS, Sigma-Aldrich, USA) in HEPES buffer along with 849*.*5 µL was added to the mix and the solution was vortexed for 10 s. This was followed by adding 61*.*5 µL N-[3-(dimethylamino)propyl]methacrylamide (DMAPAA, Poly sciences Inc., USA) to the pregel solution vial and vortexing for another 10 s. Lithium phenyl-2,4,6-trimethyl-benzoyl phosphinate (LAP, Sigma Aldrich, USA) was used as a UV-initiator for free radical polymerization. For this purpose, 154*.*8 µL of a 4 wt% LAP solution in HEPES buffer was added to the pregel solution. Finally, the pregel solution was degassed with argon gas for 5 min. The UV exposure time for GH hydrogels was 25 seconds. The conditioning procedure was performed with at least three cycles (2 h per half cycle) of switching between 1XPBS and ¼XPBS.

*Dual-responsive hydrogel (DH):* Temperature and salt-responsive hydrogels were based on copolymerizing N-isopropylacrylamide and sulfonic acid into a hydrogel. These dual-responsive hydrogels showed good thermo-sensitivity to temperature and solutions of sodium chloride in previous work [49]. The pregel solution was prepared by mixing 1097.65 mg of N-isopropylacrylamide (NiPAAm, Sigma Aldrich, USA) with 62.18 mg of 2-acrylamido-2- methylpropane sulfonic acid (AMPS, Sigma Aldrich, USA). Then, 61.67 mg of BIS as well as 8.27 mL DI water were added followed by mixing. 40 mg of the photo initiator LAP was added to the solution. The pregel solution was kept at predominantly basic conditions by adding 0.6 mL of 1 M sodium hydroxide (NaOH, Thermo Fisher Scientific, USA). Finally, the pregel solution was degassed with argon gas for 5 min. For DH hydrogels, a UV exposure of 90 s was applied in three 30 s intervals with 5 s breaks in between to prevent heat up. Microfluidic channels that contained DH hydrogels were flushed with DI water instead of PBS to remove uncured reagents. The conditioning procedure was performed with at least three cycles (2 h per half cycle) of switching between 1XPBS and ¼XPBS.

**S3. Performance Evaluation for the Image Processing Algorithm**

The performance metric used to evaluate the performance of the CV was the Jaccard index. This metric is commonly used for segmentation and detection algorithms, as it evaluates the overlap between the area manually annotated as hydrogel and the area predicted by the CV algorithm. This metric can be calculated as


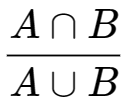


where A and B are the manually annotated and predicted hydrogel disks, respectively. Two studies were conducted to evaluate the performance and robustness of the algorithm to varying parameters and conditions, using the data from the glucose-sensitive hydrogel, shown in Figure 4 b of the main text. First, the impact of the number of images used to train the random forest classifier was evaluated. This evaluation considered a training dataset containing 1 to 4 images. The resulting models were evaluated using a random set of images containing 10 to 40 images, an order of magnitude more than the size of the training dataset for each model. This evaluation showed that the mean Jaccard index improves in correlation with the number of images used during training (see Fig S1). The results also show that the performance of the CV algorithm is acceptable (>90% Jaccard Index) even when using only one training image, suggesting that the algorithm is robust to changes in the random forest classifier. The underlying reason for such behavior is that the classifier is solely used to detect the edge of the hydrogel disk, the detected edge is then processed by a Hough transform which does not require a perfect edge to reliably estimate the radius and center point of the hydrogel disk. The results support that the number of training images (n = 3) used for all hydrogel characterization experiments is adequate.


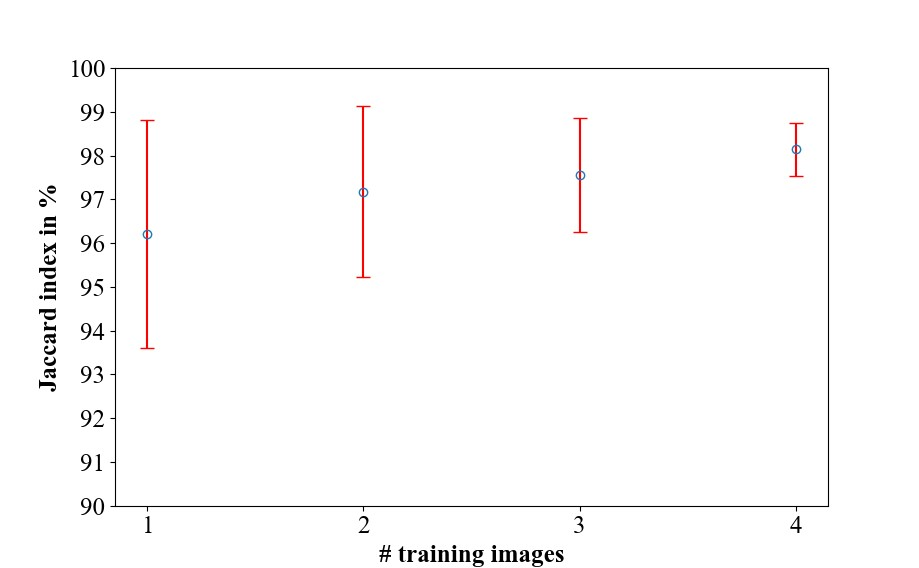


**Fig. S1. Impact of training set size on the performance of the CV algorithm.**

As a second part, the study was extended to verify that the performance of the CV algorithm is comparable when evaluating the surface area of different hydrogel disks in the microfluidic channel. The CV algorithm was designed considering that the acquired imaged is first cropped to extract the single hydrogel disks. This initial cropping step was performed due to the varying light conditions found across the acquired image, which increases the complexity for a generalized model to efficiently detect the edge of different hydrogel disks. However, to evaluate whether the CV algorithm can perform adequately across different hydrogel disks, the models (previously trained for each disk of the GH) were evaluated using 30 random images. The Jaccard Index was then calculated, resulting in an acceptable performance for all hydrogel disks as shown in Table S2.

**Table S2. Performance values for all hydrogel disks inside the evaluated microchannel.**

| Hydrogel Disk # | Jaccard Index (%) |
| --- | --- |
| P1 | 98.0 ± 0.8 |
| P2 | 99.0 ± 0.9 |
| P3 | 98.0 ± 0.6 |
| P4 | 97.0 ± 0.6 |

**S4. Response of multiple GH disks in a channel to various glucose concentrations.**

In this experiment, the response to a variety of glucose concentrations in 1XPBS was studied. The glucose concentration increments were chosen to be in the physiologically relevant range. Figure S2 shows the swelling response for three half cycles of glucose variation and three hydrogel disks (300 µm in diameter) obtained with the optical evaluation setup and reflects the raw data for Fig. 5. The measurements show a significant shrinking of the hydrogel structures with increasing glucose concentration, which is consistent with the results of previous work on hydrogel-based glucose sensors [18].

| 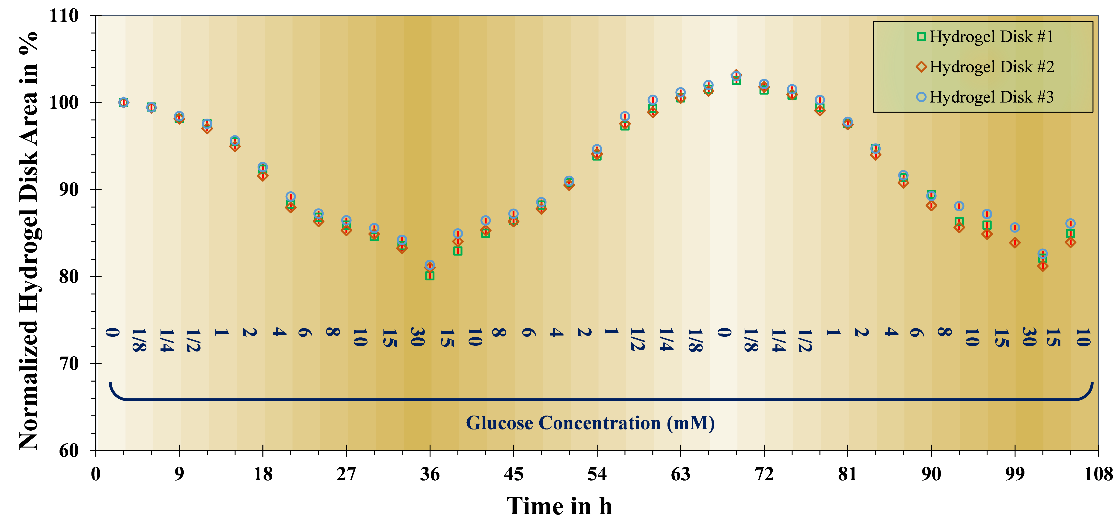 |
| --- |
| **Fig. S2. Steady-state values of the top surface area of three hydrogel disks in a single microfluidic channel over time (fabrication diameter: 300 µm). The steady-state value was obtained from images after letting the hydrogel equilibrate for 3 h. The data is normalized to the average surface area at 1XPBS.** |

**S5. Time Resolved Salt and Temperature Response of DH Disks**

Multiple cycles of NaCl solutions in DI water were introduced to a microchannel to record the dynamic response of DH hydrogels. Figure S3 shows the swelling response of the hydrogel disks (300 µm in diameter) as a function of sodium chloride concentration and time. The measurements show good repeatability. Swelling and shrinking times (τ_90_) were measured as 9.0 min and 3.5 min, respectively.

| 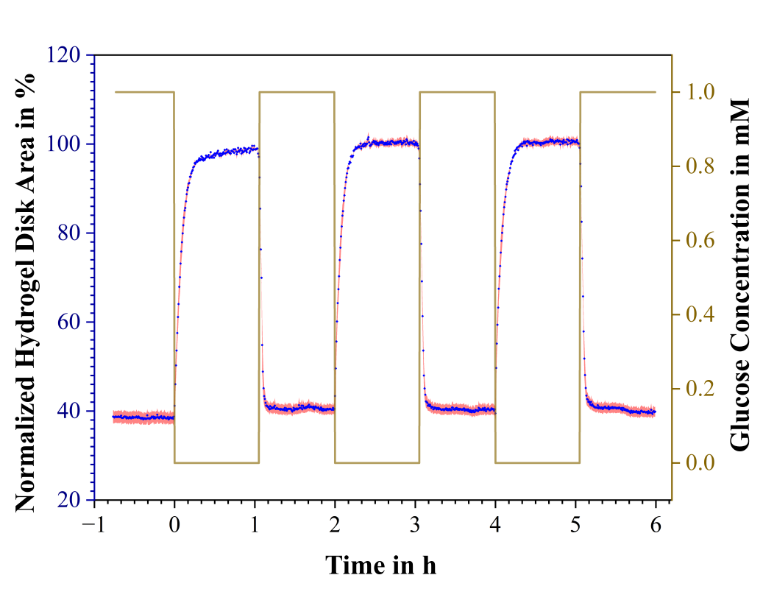 |
| --- |
| **Fig. S3. Time-resolved swelling response of hydrogel disks (diameter: 300 µm) to NaCl concentrations. Error bars show the standard error based on three hydrogel disks. The hydrogel top surface area was normalized to the value in 1XPBS.** |

Figure S4 shows the time-resolved data for multiple steps of temperature from 15°C to 40°C and reflects the raw data for Fig. 8. It can be seen that upon temperature change the hydrogel disks reached their steady state swelling degree within a few seconds.


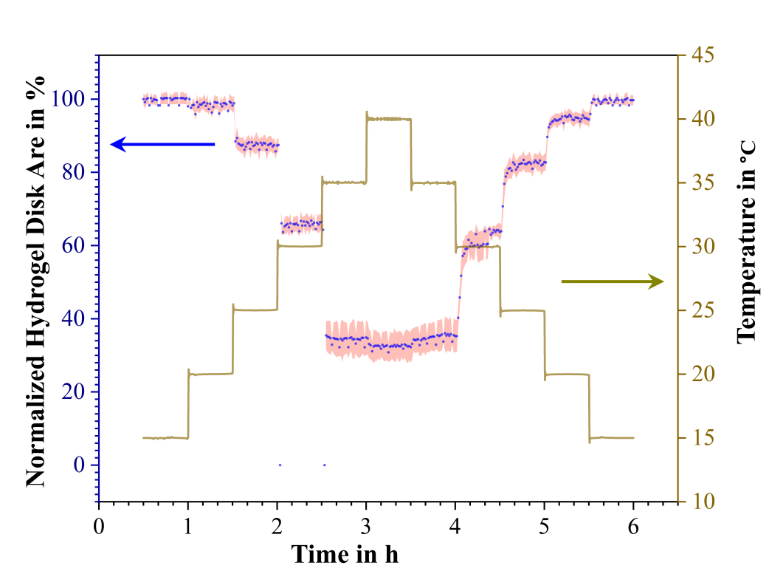


**Fig. S4. Time-resolved swelling data of three hydrogel disks (diameter: 300 µm) inside a microchannel with respect to temperature changes. The microchannel was operated with DI water and the temperature was adjusted with a temperature controller (uncertainty:** ±**0.1 K). Error bars represent standard error.**

**S6. Reliability of the Optical Platform, Microchannel and GH for Long-Term Studies.**

In this experiment, a long-term measurement with multiple cycles of 1XPBS and 10 mM glucose solutions was conducted. Figure S5 shows the steady-state values in 1XPBS of the GH hydrogel over the course of a month. The swelling degree was studied for ten days and then the microchannel was kept sealed for another ten days in 1XPBS. The third stage of the study involved introducing multiple cycles of the glucose and PBS again until the study was completed. The aim of the study was to evaluate the reliability of setup components and the stability of the baseline hydrogel area in 1XPBS. The average area during 30 days in 1XPBS was 98.90% of the initial area and as a measure of uncertainty, the standard deviation was 0.38%.


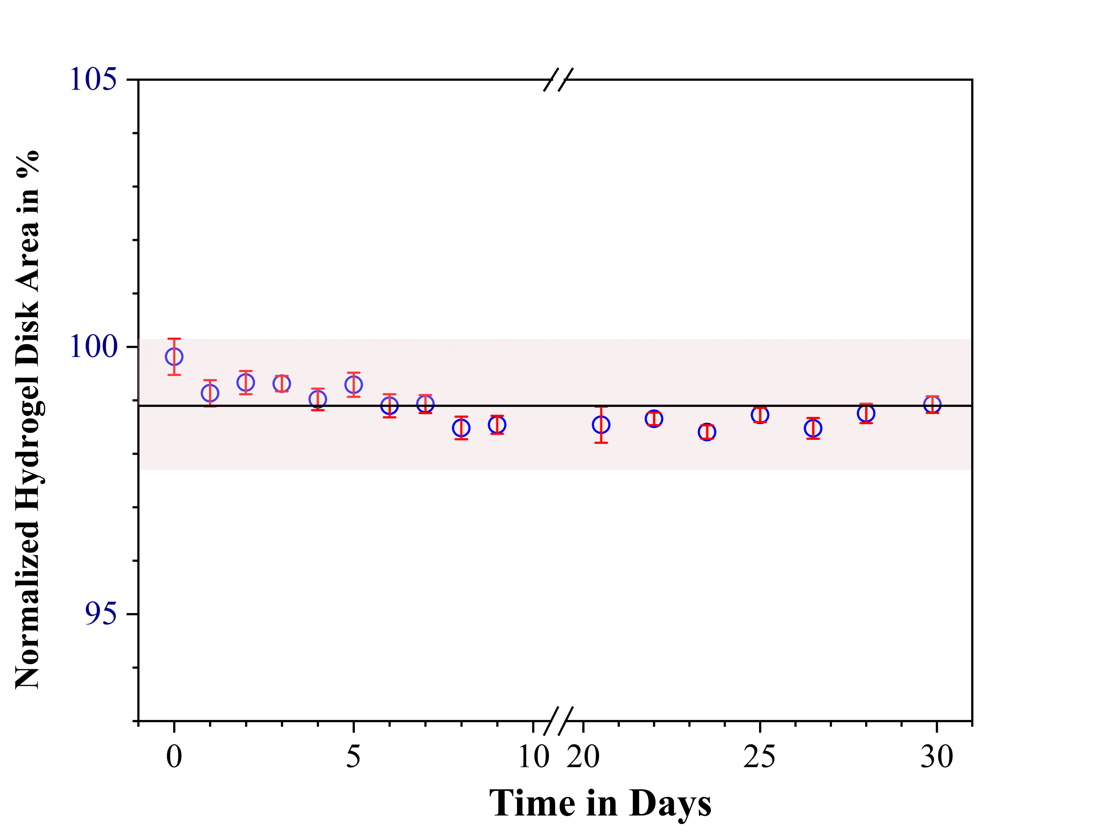


**Fig. S5. Equilibrium state swelling data of three hydrogel disks (diameter: 300 µm) inside a microchannel in 1XPBS. The microchannel was operated with automated valves to introduce periodic pulses of 10 mM glucose and 1XPBS each day. Error bars represent the standard error. The black line shows the average hydrogel disk area during the 30 days evaluation period and the red region three times the standard deviation of the average hydrogel disk area in 1XPBS during the entire test period.**

**S7. List of Parameters for Image Processing Pipeline.**

**Table. S3. Preprocessing parameters for image pipeline**

| **Parameter** | **Macro Command for Python^®^** |
| --- | --- |
| Crop | imp.setRoi(y1, y2,x1, x2); |
| Type 8-bit | IJ.run(imp, "8-bit", ""); |
| Scale (1/2 x) | imp = imp.resize(0.5×cropped image length , 0.5×cropped image width, "bilinear"); |
| Enhanced local contrast (CLAHE) | IJ.run(imp, "Enhance Local Contrast (CLAHE)", "blocksize=256 histogram=512 maximum=5 mask=*None*"); |

**Table S4. Postprocessing parameters for image pipeline**

| **Parameter** | **Macro Command for Python®** |
| --- | --- |
| Median | IJ.run(imp, "Median...", "radius=1.5"); |
| Scale (2x) | imp = imp.resize(2×segmented image length , 2×segmented image width, "bilinear"); |
| Binary Mask | IJ.setAutoThreshold(imp, "Default no-reset");  Prefs.blackBackground = true;  IJ.run(imp, "Convert to Mask", ""); |


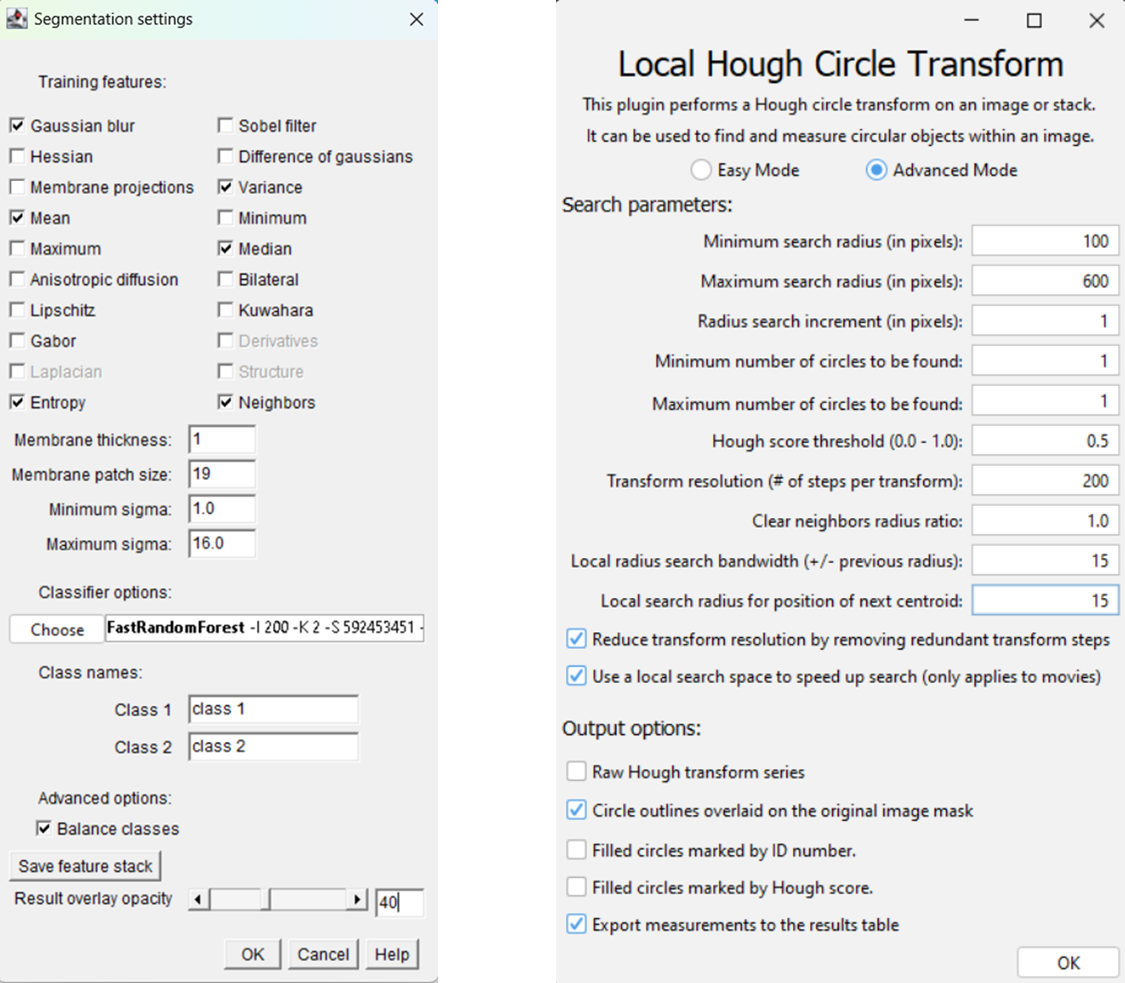


**Fig. S6. List of parameters and values for the classifier (left) and the Hough process (right).**

**S8. Top View images for select samples for Figure 5-8.**

Figures S7-S11 show the corresponding images for the swelling response of one hydrogel sample for each study at different test steps. All studies were carried out using a UV mask with 300 µm in diameter resulting in a disk size close to 300 µm in 1XPBS.


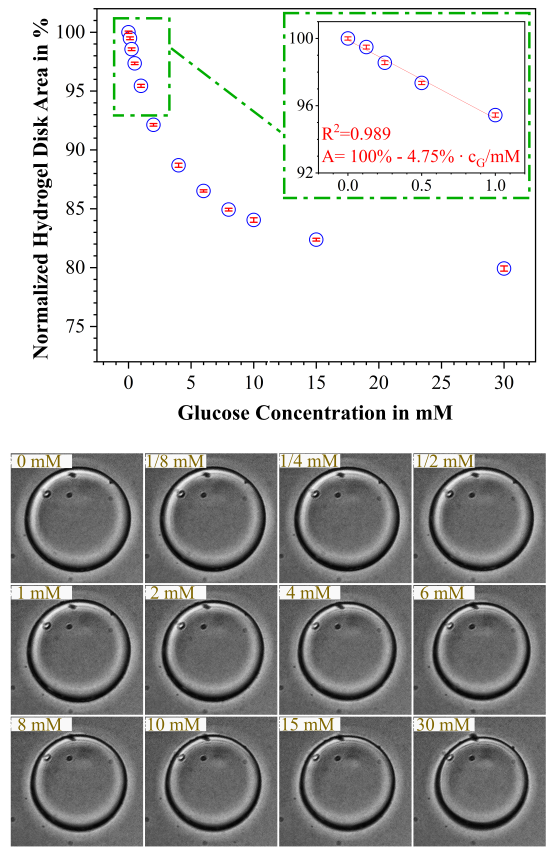


**Fig. S7. Steady-state values of 300 µm (in 1XPBS) hydrogels’ cross-sectional disk area normalized to the disk area in 1XPBS. Swelling data from the images of each step’s last 10 min were averaged. Error bars represent the standard deviation when considering the swelling response of all three evaluated hydrogel disks in the field of view of the camera sensors. Images for one of the hydrogel disks at the end of each test step is also shown.**

**
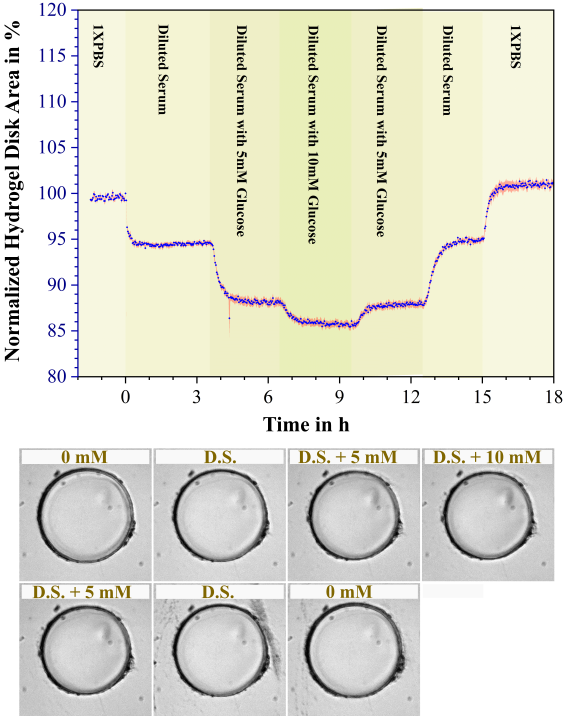
**

**Fig. S8. Glucose response of GH in serum. Time response of glucose-responsive hydrogel disks (diameter: 300 µm in 1XPBS) towards different concentrations of glucose in diluted serum (n = 4) as well as the baseline area in 1XPBS is plotted. The red shaded area represents the associated standard error at each time point. Images for one of the four hydrogel disks at the end of each test step is shown.**

**
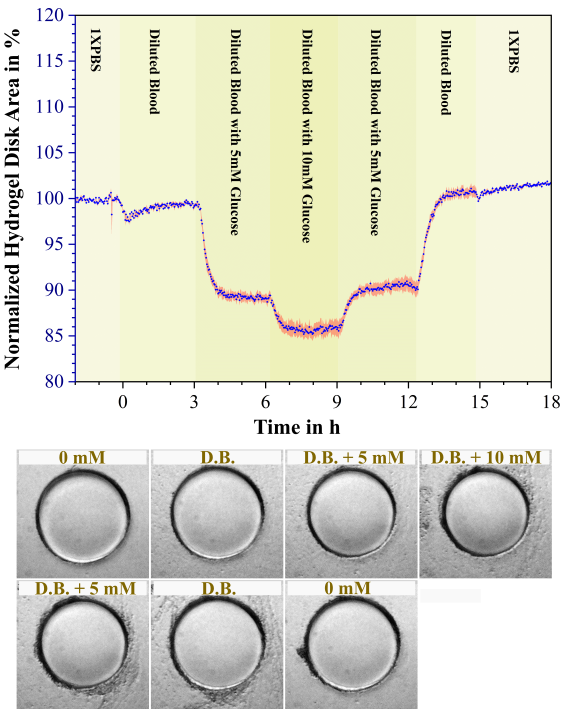
**

**Fig. S9. Glucose response of GH in human blood. Time response of glucose-responsive hydrogel disks (diameter: 300 µm in 1XPBS) towards different concentrations of glucose in diluted blood (n = 3) as well as the baseline area in 1XPBS is presented. The red shaded area represents the associated standard error at each time point. Corresponding Images for one of the three hydrogel disks at the end of each test step is presented.**

**
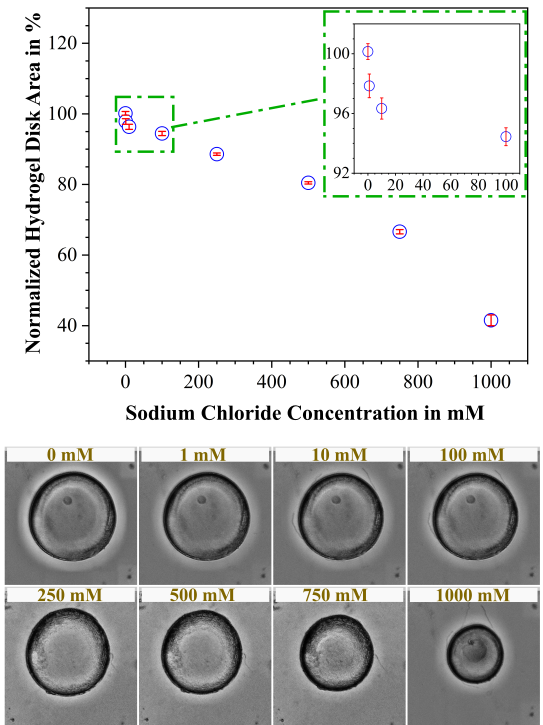
**

**Fig. S10. Steady-state swelling data for three hydrogel disks (diameter: 300 µm in 1XPBS) as a function of NaCl_(aq)_ concentration. The swelling data and the standard error at each step were averaged from the images of three disks during the last 10 min. Images corresponding to one of the three hydrogel disks at the end of each test step is presented.**

**
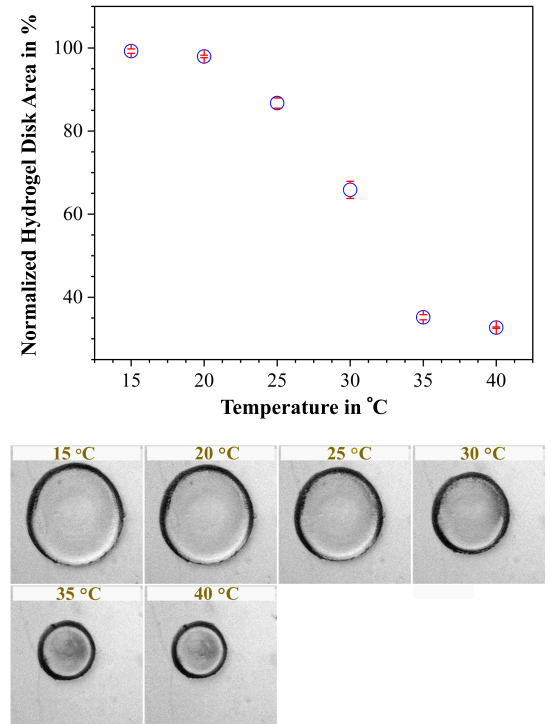
**

**Fig. S11. Steady-state swelling data of hydrogel disks (diameter: 300 µm in 1XPBS) inside a microfluidic channel as a function of temperature. DI water was passed through the channel while adjusting the temperature (uncertainty:** ±**0.1 K). The change between temperatures takes place in less than 15 seconds, due to the active heating and cooling by means of the Peltier element. Error bars represent the standard error for three hydrogel disk measurements during the last 10 minutes of each analyte solution cycle. Images correspond to one of the hydrogel disks at the end of each test step is presented.**
